# Networking-Aware IoT Application Development

**DOI:** 10.3390/s20030897

**Published:** 2020-02-07

**Authors:** Arne Bröring, Jan Seeger, Manos Papoutsakis, Konstantinos Fysarakis, Ahmad Caracalli

**Affiliations:** 1 Siemens AG, Corporate Technology, 81739 Munich, Germany; 2 Chair of Networking Architectures and Services, Technical University Munich, 80333 Munich, Germany; seeger@in.tum.de; 3 School of Mathematics, Computer Sciences and Engineering; City University of London, Northampton Square, Clerkenwell, London EC1V 0HB, UK; Emmanouil.Papoutsakis@city.ac.uk or; 4 Foundation for Research and Technology Hellas, Institute of Computer Science; N.Plastira 100, 70013 Heraklion, Crete, Greece; 5 Sphynx Technology Solutions AG, 6300 Zug, Switzerland; fysarakis@sphynx.ch; 6 EURECOM, Campus SophiaTech, 06410 Biot, France; ahmad.caracalli@eurecom.fr

**Keywords:** IoT, SDN, semantic models

## Abstract

Various tools support developers in the creation of IoT applications. In general, such tools focus on the business logic, which is important for application development, however, for IoT applications in particular, it is crucial to consider the network, as they are intrinsically based on interconnected devices and services. IoT application developers do not have in depth expertise in configuring networks and physical connections between devices. Hence, approaches are required that automatically deduct these configurations. We address this challenge in this work with an architecture and associated data models that enable networking-aware IoT application development. We evaluate our approach in the context of an application for oil leakage detection in wind turbines.

## 1. Introduction

Today, building IoT applications is more and more supported by tools as well as by standardized activities for networking, accessing, or controlling devices. Standards (e.g., W3C Web of Things [1], OPC UA [2], or OneM2M [3]) allow the reliable development of integration and interaction mechanisms between IoT devices or platforms. No-/low-code tools enable the easy composition of devices and their functionalities to combine them on a higher level to IoT applications. For cloud and mobile environments, examples for such tools are “If This Then That” (http://ifttt.com) or Mendix (https://mendix.com). For the device-level, an example tool is Node-RED (http://nodered.org) that supports the IoT application development with a visual flow programming approach. Facilitating the IoT application development with such tools becomes a key enabler towards an IoT app economy [4] with novel business models.

While the composition of IoT application development is well supported and is becoming easier today, the focus is solely on the flow and business logic of the application. The network between IoT devices and platforms is typically assumed as existing and not considered needing to be adjusted or managed by such IoT tools. Instead, the network is engineered separately and no integrated view on the application/network interplay is given. This is an issue as the network configuration underlying an IoT application can be crucial for its successful execution. An example is the case of an intrusion detection application with three devices involved: (1) a surveillance camera streams their video feed to (2) an artificial intelligence (AI) analytics service running on an IoT/edge device that triggers (3) an alarm hosted by a third device. In such an example, the IoT application is relying on the network to provide the required bandwidth for multiple HD camera feeds as well as be reliably available for sending out the alarms. This is getting particularly challenging if multiple IoT applications are implicitly relying on hard quality of service (QoS) constraints of the network.

In this paper, we present an integrative approach that allows the composition of IoT applications in conjunction with semantically-enabled requirement definitions towards the network. We design an architecture and implement its components that can be utilized to visually compose IoT applications and define application-level constraints towards the network. We enable the automatic translation of these application-level constraints into patterns regarding security, privacy, dependability, and interoperability (SPDI) aspects of the network. As input for a rule engine, these patterns can be automatically monitored and enforced through our approach. As compared to traditional SDN network setups, this enables low-code and dynamic management of the underlying network without having to manually manage the configuration overhead of the Software-defined Networking (SDN) Controller. We thus provide mechanisms to develop distributed IoT applications under consideration of QoS constraints and for the flexible enforcement of these constraints. For the basis of the tool to create IoT applications, we utilize Node-RED, as it gains more and more traction in the industry, while still being open, extendable, and reliant on a large open source community. We abstract from the network and device specific properties by using the semantically-enriched format of Thing Descriptions [5], which are discoverable via a Thing Directory.

We demonstrate our developed solution via an application that consists of multiple IoT devices and services to detect oil leakage in wind turbines. As a proof of concept, this IoT application integrates a camera that transmits its video stream to a second device for AI-based image classification and a third device is triggered to stop the wind turbine in case leaked grease is detected. We evaluate our approach with this application concerning the network utilization and performance of the semantic reasoning.

This work describes the latest findings on our research agenda to enable IoT applications as distributed choreographies. Our agenda started with the definition of the *Recipe* concept as template for IoT applications [6], continued by our approach for runtime management of such recipes [7], the dynamic and resilient management of IoT applications [8], and rule-based configuration of networks underlying the IoT application [9]. This work combines the results of these previous works and goes beyond by investigating, implementing, and evaluating an approach that automatically translates application-specific SPDI and QoS constraints into executable patterns/rules as well as their actual monitoring and enforcement.

The remainder of this paper is structured as follows. Section 2 presents relevant works from the field of service and device composition using semantic descriptions as well as software defined networking to enforce networking QoS. Section 3 outlines the architecture of our approach. Section 4 describes the developed model for the design of IoT application lows as well as the pattern language for IoT orchestrations. Section 5 describes the mechanisms for the automated translation of application flows into network configurations, for automated processing of these configurations, and for automatically configuring the SDN. Section 6 presents the example of the oil leakage detection application and evaluates the approach. Section 7 draws conclusions and lessons learned from the results of this work and outlines derived future research directions.

## 2. Background and Related Work

IoT applications and systems are composed of IoT devices and their functions. *Service composition* addresses the difficulties of finding and linking devices and functions. Orchestration and choreography are the two types of service compositions distinguished literature [10]. Orchestration is centered around a controlling service steering the interplay of the other involved services. Choreography distributes this control, i.e., each service acts upon its on knowledge or configuration in the interaction with others. In both cases, compositions of services can be formally described by relying on the description of each service. This can be similarly done for the composition of IoT devices and their functions.

The semantic enrichment of service descriptions makes their discovery more powerful. This allows resources to be identified in conjunction with expanded semantics. The design of services can also be progressed by automatically identifying matching services that can communicate by utilizing their semantic descriptions. WS-BPEL, which is widely supported in practice, is a standard for *syntactic* Web service orchestration. SOA4All was an example of a project dealing with issues related to the semantic network orchestration of WSDL / SOAP-based web services [11], while RESTful services can be semantically described with e.g., hRESTS [12] or RESTdesc [13]. The W3C Web of Things (WoT) initiative specifies a semantic description format dedicated for IoT devices and their functions: the Thing Description (TD) [5], which contains metadata about the device and possible interactions with it. In [14], the applications are described as sets of semantic rules. However, this approach does not take into account definition of QoS constraints. Also, visual programming support to facilitate application development is not provided.

There are a range of frameworks for system composition to design automation activities for new services. A systematic survey on the composition of cloud-based QoS-aware web services can be found in [15], however, this study concentrates on cloud-level platforms, while we focus on localized, edge-level composition of IoT devices and also specifically considering QoS constraints. Another example for a QoS-aware approach for service composition is described by Mokhtar et al. [16] and also based on matching service semantics. Also in [17], a framework is presented that qualifies QoS capabilities, in this case through the management of trust. In [18] a dependable platform for the composition of services is presented. The previous work has in common that QoS constraints are defined at service level, while the utilized network is not considered. On the other hand, we intend to also explicitly consider the network and configure, monitor, and ensure QoS requirements on that level as well.

Software-defined networking (SDN) centralizes routing decisions in a central controller and thus provides finer-grained control over network settings, as compared to traditional distributed approaches [19]. The controller separates the data and control planes from each other, allowing routers and switches to forward data based on a global view of the network. Protocols such as OpenFlow [20] allow routers and switches to enforce QoS constraints using queues and meters.

Traditional protocols for network-wide QoS control are Differential Services (DiffServ) [21] and Integrated Services (IntServ) [22]. DiffServ describes coarse-grained traffic classes, and relies on a decentralized configuration of network elements. This class-based approach is not able to differentiate the many different requirements that automation applications can require. IntServ provides a finer-grained approach, but is not widely supported by consumer hardware, and has scalability issues in larger systems. Both protocols do not provide a centralized view of the system, and cannot support the fine-grained control realizable with a central controller with global knowledge of the network.

SDN is often complemented by Network Function Virtualization (NFV), which further shifts the network management towards software. Separate Virtual Network Functions (VNFs) can implement network functions such as firewalls or load balancers, while being virtually executed in a containerized environment that is scalable to the actual demand, e.g., to support tactile internet [23]. Based on an SDN-enabled network, [24] presents an approach for the optimal allocation of such VNFs; similar to the allocation of application tasks as described in [25]. Such an optimal allocation approach could be implemented by the rule system proposed in this work, particularly, to extend the efficiency of running applications.

Various research works focus on the enforcement of QoS parameters via SDN protocols. Naman et al. [26] propose an API for providing visibility into the network state, and implement an SDN-assisted congestion control algorithm for satisfying demands for low latency and high bandwidth. Akella et al. [27] work on guaranteeing bandwidth allocations for prioritized cloud users. Kucminski et al. [28] present a QoS-based routing scheme for prioritizing important traffic over less important traffic. Li et al. [29] take a step back and try to identify application classes at the SDN controller. Different QoS classes are then defined for the different types of applications. Guck et al. [30] implement and evaluate a network model for guaranteed latency with a reasonable processing cost. Gorlatch et al. [31] translate high-level QoS requirement into low-level SDN configurations for optimizing response-time in real-time interactive applications.

However, no systematic integration with application development tools has been attempted so far. Our aim is the creation of a system for the integrated development of application and network constraints.

## 3. An Architecture for Network-Aware IoT Applications

We define the term *IoT application* as a workflow of interacting services (e.g., sensing, acting, storing, or computing) offered by different IoT devices. Thereby, multiple IoT applications can be executed within an *IoT environment*, a physical space that comprises several networked IoT devices. Today, when new devices and applications are added to such an IoT environment, there is manual integration effort, e.g., the device’s parameterization needs to be adjusted or a centralized network controller may need to be reconfigured. Manually designing and deploying such compositions of IoT applications can be time-consuming and error prone [10].

To support the design of IoT applications, there are commercially available systems such as “If This Then That” (IFTTT) (http://ifttt.com). The platform provides a simple interface to create and execute cloud-centralized orchestrations of IoT service choreographies, however, it lacks systematic engineering support [32]. The Node-RED (http://nodered.org) tool focuses also on usability and therefore follows a visual programming approach. Its browser-based editor can be easily used to connect IoT devices, APIs, and online services through an interactive drag and drop and wiring to an application flow.

A shortcoming of Node-RED is that developed applications cannot be executed in a decentralized way, i.e., all program logic designed in a flow is executed locally on one machine—even when defining flows in separate sub-flows or on different tabs. It is possible to view multiple Node-RED instances at a central machine and connect them via communication protocol nodes, e.g., for MQTT, UDP, or HTTP. However, managing such connections manually between the Node-RED instances of the various involved devices would be complex and error-prone. This is where Distributed Node-RED (DNR) [33] fills a gap and enables the definition of distributed flows by allowing the ability to define on which device each node runs. Thereby, DNR provides a tool to configure the IoT application centrally and automatically communicate the design and changes of the flow to the involved devices. The communication between the now distributed nodes is realized based on a MQTT broker contained in the DNR distribution, i.e., all communications, not only management of flow handling but also data transfer between nodes, is exchanged via this MQTT broker. In this paper we build up on DNR, but extend it to allow direct communication (circumventing the MQTT broker, e.g., via UDP) and enable the underlying network configuration through semantically defined QoS requirements.

In our previous work [6,7,8], we introduced the *recipe* concept to represent the design of an IoT application, i.e., a composition of services from IoT devices, separate from its implementation. A graphical tool allows the ability to define and instantiate the recipe. The user is supported during the instantiation of the recipe by narrowing down matching recipe ingredients through semantic subsumption reasoning. Then, we enabled the distributed execution of instantiated recipes in [7]. Our approach went beyond the concepts of [33] by introducing mechanisms for fault tolerance and failure detection (see [8]) as needed by critical automation systems.

Missing in the above works and tools, is the representation of the network when creating IoT applications. In case of Node-RED, the developer is designing the data flow to create an IoT application. Generally, this focus on the business logic is important for application development. However, for IoT applications in particular, it is crucial to consider the network, as they are intrinsically based on interconnected devices and services. Often, the user/developer does not have in depth expertise in configuring the network and physical connections between the involved IoT devices. Hence, approaches are required, which automatically deduct these configurations. To address this challenge, we have designed and implemented the components of an architecture with associated data models (Section 4) that allow this integrative IoT application development.

**Figure 1:**
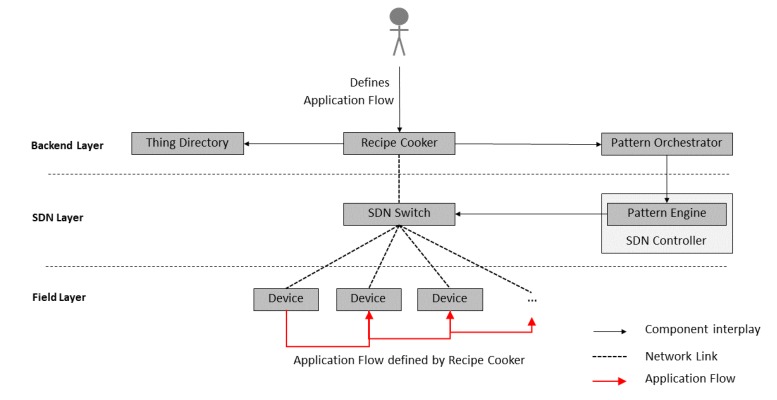
Overview of architectural components and their interplay.

Figure 1 shows the key components of this architecture and their interplay. At the center is the *Recipe Cooker*, which is responsible for creating IoT applications that reflect user requirements on different layers (cloud, edge, and network), transforming recipes into executable rules. For this work, the Recipe Cooker component has been re-implemented and based on Distributed Node-RED [33].

In order to receive semantic descriptions of available IoT devices and their functions, the Recipe Cooker connects to the *Thing Directory*. This component hosts Thing Descriptions (TDs) of registered IoT devices and can be used to browse and discover a thing based on its registered TD. The Thing Description model and serialization format are conform to the W3C definitions [5]. The directory can be used to browse and discover Things based on their TDs. This includes Searching for a thing based on its metadata, properties, actions, or events; as well as creating or deleting a thing’s TD or updating an existing one.

The application developer creates an IoT application using the Recipe Cooker. The application is represented as an orchestration pattern that comprises application-level networking constraints and follows a semantically-defined grammar (Section 4.3). This pattern-based application definition is transmitted to the *Pattern Orchestrator*, which is responsible for the automated coordination and management of patterns and their deployment. Next, this component converts the received patterns to Drools rules, which are distributed as facts to the relevant Pattern Engine.

The *Pattern Engine* component is incorporated with an SDN controller. It allows the insertion, modification, execution, and retraction of patterns at design or runtime of the SDN controller. Continuous reasoning through pattern matching ensures the secure, privacy-aware, dependable, and interoperable operation of the network and the IoT application running on top of it. The Pattern Engine is based on a rule engine, which needs to be able to express design patterns as production rules to enable reasoning. Hence, the rule engine is based on the Drools rule engine [34] that supports backward and forward chaining inference and verification, by applying and extending the Rete algorithm [35]. The SDN controller is then configured by the Pattern Engine through the OpenFlow interface (Section 2).

## 4. Models for IoT Application Flows and Network Patterns

In the following, we present models for IoT application flows that can capture application-level QoS constraints that are to be translated into network-specific constraints (Section 4.1), an approach for designing application flows with this model (Section 4.2), as well as a model for a pattern language to monitor and enforce the QoS constraints on the network (Section 4.3).

### 4.1. Model for IoT Application Flows with QoS Constraints

Initially developed to support the semantic enablement of IoT interoperability [36], we have developed the *Recipe* model [6] that allows the compositions of *ingredients* and their interactions. Ingredients are placeholders for *offerings*, devices and services that process and transform data. Interactions describe the dataflow between these ingredients. In this work, we build up on this model for defining distributed IoT applications and the application-specific QoS constrains.

**Figure 2:**
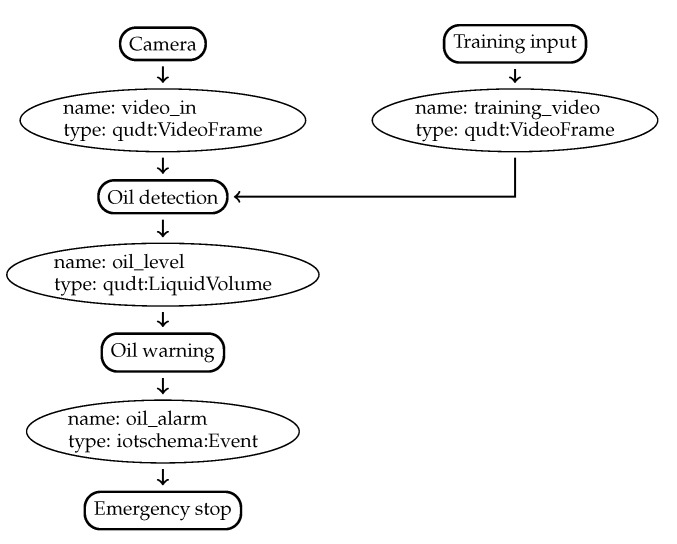
A simple oil leak detector recipe.

An example recipe is shown in Figure 2 describing a simple machine-learning based oil leak detection system. A camera records a video stream, which is passed to an oil detection component. This oil detection component derives the current oil leakage based on the image input. The amount of leaking oil is then sent to a warning component that compares the oil level to a preset or dynamic threshold. When this threshold is exceeded, an alarm message is sent to the emergency stop component to stop the machine. The type is used for matching offerings with ingredients based on the semantic type [8].

Offerings describe service or device instances, and how to access these services or devices. Offerings are specified in a semantic format by the so-called *offering description*, which is semantically-aligned with the W3C Thing Description [5] used in the Thing Directory of our architecture (Section 3). Offering descriptions contain information on the inputs and outputs of an offering as well as information on how to access the underlying service or device (providing the offering implementation). The offering contains functional as well as non-functional properties. Functional properties describe the implementation of the offering (e.g., the endpoint as well as protocol to access it), while non-functional properties describe installation-specific metadata about the offering (such as the price or location of the offering). Non-functional and functional properties thus correspond to offering *interface* and *implementation*, respectively. The offering description further contains functional properties that contain information on the types of input and output that this offering consumes and produces. Type annotations are uniform resource identifiers (URIs) referencing for example a term in the schema.org [37] or QUDT [38] ontologies. Additionally, a category can be used to classify the offering, e.g., into *smart building* or *transportation* categories.

Based on the above outline model, we have implemented application-level QoS constraints on a semantics-based platform in our previous work [9]. *Application-level* QoS constraints refer to the possibility of defining such constraints on a high-level, independent of network-level specifics. Application-level QoS constraints are thus an abstract description of an application’s network requirements. Due to being defined on the application level, such constraints are easier to define for the user, and can be stored independently of the specifics of the underlying network. An example for the use and implementation of application-level constraints can be found in [31].

We have defined a scheme for expressing application-level QoS constraints as a collection of semantic rules. Including these rules in the triple store together with the semantic models, the application-level constraints are automatically translated by the semantic reasoner of the triple store into instances of the lower-level SDN model. These instances can then be submitted as configurations to the SDN Pattern Engine.

One example for such an application-specific constraint is specifying the required bandwidth for a video stream based on the *frame rate* (*f*) of a video. This is a useful constraint in video analysis, where the algorithm requires a certain frame rate to work correctly. In the oil leakage example in Figure 2, attaching such a constraint on the link between the camera and analysis component would ensure that the input quality for the analysis component is good enough to deduce correct oil leakage information. Using application-level constraints, we can ensure the availability of bandwidth from application development onwards. For more information on the possible implementation of such constraints via a semantic reasoner, see [9].

The advantage of these application-level constraints is that they can take into account high-level parameters such as resolution or encoding efficiency, which are available in the Thing Directory. If the video format’s efficiency is e∈[−∞,1] and the video’s resolution is x×y, we can infer a minimum bandwidth with the calculation bw=(1−e)*x*y*f. The translated bandwidth constraint can then be sent to the Pattern Orchestrator, which is able to (a) monitor the fulfillment of the constraint on the network and (b) enforce the availability of bandwidth via SDN mechanisms.

### 4.2. Defining IoT Application Flows with QoS Constraints

To be able to define application flows with application-level networking requirements, we extended Distributed Node-RED (DNR) [33]. The DNR tool already provides a way to execute application flows in a distributed way, i.e., the IoT application developer can specify for each node of the application flow on which machine it should be deployed and executed. This makes DNR a powerful tool for realizing edge computing [39] applications.

**Figure 3:**
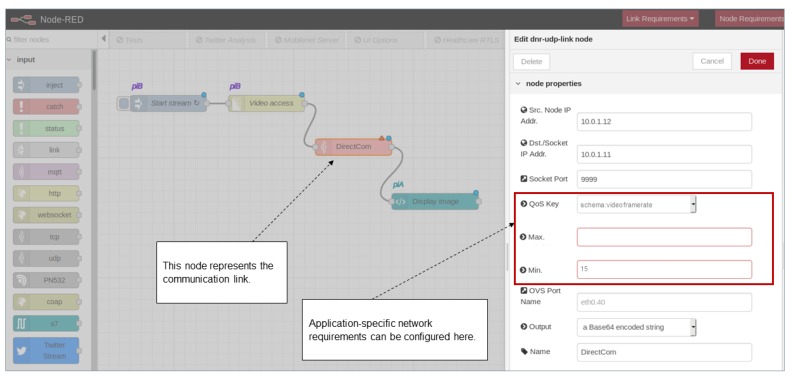
Extended Distributed Node-RED (DNR) to allow the specification of quality of service (QoS) constraints from application perspective.

**Figure 4:**
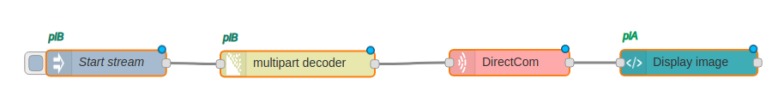
Live video transmission flow.

In Figure 3, the DNR editor is shown and a simple application flow (shown in more detail in Figure 4) is implemented that consists of four nodes transmitting a live video between two Raspberry Pi devices. Labeled with ’piB’, the *start stream* node and *multipart decoder* node (for decoding the video stream from a connected camera) are running on Raspberry Pi B. Similarly, the *display image* node is labeled with ’piA’, which means that it is running on Raspberry Pi A. We could already connect the *multipart decoder* node and the *display image* node to create a distributed flow between Raspberry Pis A and B. However, with DNR only, no further specifications for the underlying networking can be made. Hence, we developed the *DirectCom* node, which is representing the network connection (see Figure 4).

The main functionality of the DirectCom node is to create a UDP link between the source node on the left and destination node on the right. Using only the DNR without this extension, all communication (even the video data between the two nodes) happens via an MQTT server running in the background of DNR. The DirectCom node is running instances on all involved machines of the cluster (here: Raspberry Pi A and B). It launches a UDP server on the machine of the destination node and a UDP client on the machine of the source node, in order to transmit all incoming data from the source node (here: multipart decoder) to the UDP server node. In response, the UDP server forwards the received data to the next node (here: display image).

Figure 3 shows the configuration of the DirectCom node. Besides defining the IP addresses of source and destination, the socket port number of the UDP server, and the output data format (Buffer, String, or Base64 encoded string) have to be specified. The QoS key text field in the dialog of Figure 3 then allows us to define application-level QoS constraints to be applied for this specific communication link. From a drop-down menu, terms that represent application-level QoS constraints can be selected. Here, ’schema:videoframerate’ (set to a minimum of 15 frames per second) is provided to automatically translate the frame rate requirement of the application into a bandwidth constraint (Section 4). To integrate with an existing ecosystem we aligned our terms with the existing vocabulary schema.org [40].

### 4.3. Pattern-Driven Property Modeling and Management

In addition to facilitating the user-friendly definition of IoT applications and their orchestrations, an ever-present need is to monitor and enforce the desired properties that said applications must maintain. To this end, the work presented herein adopts a pattern-driven approach. Patterns are re-usable solutions to common problems and building blocks to architectures and in the context of this work they are used to encode proven dependencies between security, privacy, dependability, and interoperability (SPDI) as well as QoS properties of individual smart objects and corresponding properties of orchestrations (composition) involving them. The encoding of such dependencies will enable: (i) the verification that a smart object orchestration satisfies certain SPDI and QoS properties, and (ii) the generation (and adaptation) of orchestrations in ways that are guaranteed to satisfy required SPDI properties.

This pattern-driven approach, as recently presented in [41,42,43], is inspired from similar pattern-based approaches used in service-oriented systems [44,45], cyber-physical systems [46], and networks [47], while covering the intricacies of IoT deployments and more properties in addition to Security, and also providing guarantees and verification capabilities that span both the service orchestration and deployment perspectives.

**Figure 5:**
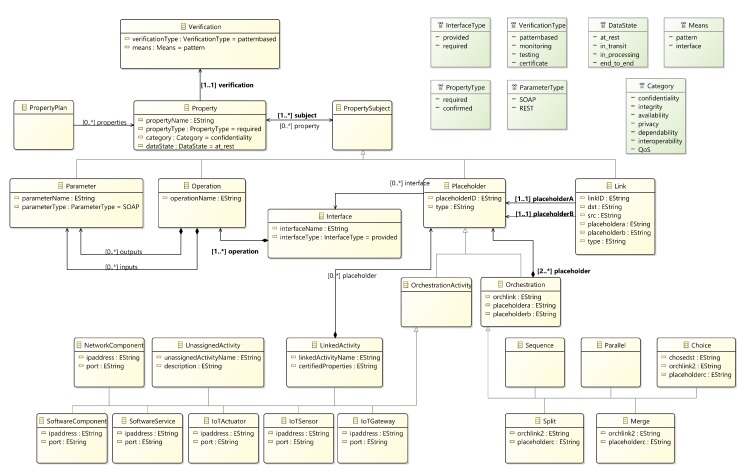
IoT orchestrations system model.

To enable the above approach, it is necessary to develop a language for specifying the components that constitute IoT applications along with their interfaces and interactions. In this context, the definition of the various functional and non-functional properties of IoT components and their orchestrations is required in the form of a model. The defined model appears in Figure 5, and is presented in detail in [41]). A model with such characteristics effectively serves as a general “architecture and workflow model” of the IoT application.

Once defined, this model is used to derive a language which will allow the definition of pattern rules and facts which, consequently, enable the reasoning required for verifying SPDI and QoS properties in specific IoT applications and subsequently enable different types of adaptations. The derived language for defining IoT application models adopts an orchestration-based approach. An orchestration of activities may be of different types depending on the order in which the different activities involved in it must be executed (e.g., sequence, parallel, choice, and merge). Moreover, an orchestration involves orchestration activities. The implementation of an activity in an IoT application orchestration may be provided by a software component, software service, network component, an IoT sensor, actuator or gateway, as well as a sub-orchestration of IoT application activities of the previous types). These types of IoT application activity implementers are grouped under the general concept of a placeholder, which is accessible through a set of interfaces.

Based on the above, language constructs are used to define an orchestration pattern. A textual representation of the model in the form of an EBNF [48] grammar is used as input to the Eclipse ANTLR4 [49] plugin for the creation of a lexer and parser. In this way, any input can be checked for compliance with the defined grammar. For the sake of brevity, only a sample for the definition of a *Placeholder* is presented in Listing 1.

## 5. Implementation

In the following, we describe the mechanisms for automated translation of application flows into network configurations (Section 5.1) as well as a mechanism for the monitoring of these configurations (Section 5.2).

Listing 1: Pattern Language Grammar Snippet

placeholder
   :   placeholdertitle OPEN_PAREN placeholderid COMMA interfacename (COMMA interfacename)*
        COMMA propertyname (COMMA propertyname)* CLOSE_PAREN
   |   orchestration
   |   orchestrationactivity
   ;
			


### 5.1. Translation of Application Flows into Network Configuration

To monitor and enforce QoS properties using the Pattern Engine, we must transform the IoT application as defined in the Recipe Cooker (Section 3) from an application flow into the pattern language consumed by the Pattern Orchestrator, which forwards it to the Pattern Engine to be monitored and enforced (Section 5.2). Our input is in formatted in JavaScript Object Notation (JSON), the standard Node-RED flow export format. We read this input using library functionality, and transform it into a graph. Then, we run a number of graph reduction steps while emitting pattern language elements. These steps are, in order:

1. Emit placeholders and their static properties.
2. Merging two nodes and one link into a Sequence.
3. Merging three nodes where two nodes are connected to one node into a Merge.
4. Merging three nodes where one node is connected to two nodes into a Choice.
5. Emit properties that need to be proven.

Steps 1 and 5 are only executed once, while Steps 2 to 4 are executed until they no longer change the resulting graph. Each translation step emits pattern language elements and shrinks the graph for the next transformation step. It is easy to see that each step reduces the size of the graph by at least one, as at least two nodes are merged into one. This means this algorithm is guaranteed to finish eventually.

**Figure 6:**
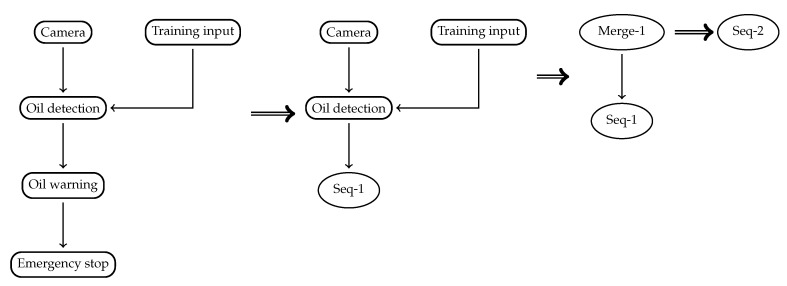
Translation steps from application graph into pattern language.

An example for the translation steps is shown in Figure 6. Before the translation, all components are translated into placeholders, software components, and hosts for communicating device information such as MAC and IP address. Additionally, we emit links between components. We have implemented this transformation in a Python (http://python.org) script using the networkx library.

Then we start the graph conversion process. In the first step, a sequence is created from two nodes, causing a Sequence node consisting of two placeholders to be created. Then, a merge is created from three nodes, causing a Merge node consisting three placeholders to be created. Finally, another sequence is created from the Merge and Sequence nodes. The graph consists of only one node, so the transformation is complete. The shortened output looks like this:



Placeholder("Camera"),
Placeholder("Oil detection"),
Placeholder("Training Input"),
Placeholder("Oil warning"),
Placeholder("Emergency stop"),
Link("Link1", "Camera", "Oil detection"),
Link("Link2", "Oil detection", "Oil warning"),
Link("Link3", "Oil warning", "Emergency stop"),
Link("Link4", "Training input", "Oil detection"),
Sequence("Seq-1", "Oil warning", "Emergency stop", "Link3"),
Merge("Merge-1", "Camera", "Training input", "Oil detection", "Link1", "Link4"),
Sequence("Seq-2", "Merge-1", "Seq-1", "Link2")
*# Static properties*
Property("Prop0", required, qosbandwidth, "11400000.0", "Camera", true),
Property("Prop1", required, qosbandwidth, "11400000.0", "Oil detection", true),
*# To-be-proven properties*
Property("Prop2", required, qosbandwidth, "4000000",..., false)
*# Added by monitoring system*
Property("Prop3", required, qosbandwidth, "11400000.0", "Link1", true)
			


Additionally, to allow the monitoring of network configurations, we add properties to the graph. These properties are either static facts about the devices (such as available link bandwidth or processing speed), or need to be proven by the Pattern Orchestration Engine (such as required bandwidth, or maximum latency). Static information is retrieved from the Thing Directory, while “to-be-proven” properties are specified in the UI. Static properties are indicated by a true in the final position, while the Pattern Engine tries to prove those properties that have a false as final parameter. To be able to prove this, a monitoring system periodically updates the properties of network, as described in the next section.

### 5.2. Automated Processing of Network Configurations

An important requirement for implementing the pattern-driven management and adaptation of IoT applications is to support the automated processing of the patterns developed using the language described in Section 4.3. To achieve this, the SPDI patterns can be expressed as *Drools* [50] business production rules, and the associated rule engine, by applying and extending the Rete algorithm [35]. The latter is an efficient pattern-matching algorithm known to scale well for large numbers of rules and data sets of facts, thus allowing for an efficient implementation of the pattern-based reasoning process. A Drools production rule has the following generic structure:



rule name <*attributes*>* when <*conditional element*>* then <*action*>* end
			


Thus, herein Drools are leveraged to encode the relation between properties in SPDI and QoS patterns in a way that allows the inference of the activity properties required of the activity placeholders present in the orchestration of said pattern in order for the orchestration to have the SPDI property guaranteed by the pattern.

The IoT application transformed into the pattern language is communicated to the Pattern Orchestrator and is fed to an ANTLR4 lexer, parser, and listener. These three programs manage to create a Drools fact, i.e., an instance of the corresponding Java class of the IoT application model, for every orchestration activity, control flow operation, or property. During this procedure, the ANTLR4 lexer recognizes keywords and transforms them into tokens. The created tokens are used by the ANTLR4 parser for creating the logical structure, i.e., the parse tree. The main functionality of the ANTLR4 listener is to become aware of the node additions in the parse tree. Whenever such an addition takes place, the listener takes information from the tokens that were used for the creation of instances of the corresponding Java classes. Afterwards, the received information is stored at the class attributes. Finally, the created Java instances are sent to the corresponding Pattern Engine as facts, where they are inserted into knowledge sessions of Drools Engine. These Drools facts are used by Drools rules, which are fired when their conditions are met.

The communication between the Pattern Orchestrator and the Pattern Engine is done through a REST API, which comprises the methods for the creation, deletion, and retrieval of facts. The request for sending a Drools fact uses the HTTP POST method and its URL is http://[PatternEngineIP]/patternengine:addFact. In the body of the request, there is a Fact object with five field names presented in Table 1 below.

When a Drools fact is received by the Pattern Engine, it is inserted in the Drools Rule Engine, part of a business rule management system (BRMS). Upon the arrival of a Drools fact, a new KIE (Knowledge Is Everything) session is created. This session is used for the insertion of the Drools fact into the working memory of Drools Rule Engine. Drools Rules are contained in the RuleBase, ready to be used. Such rules preexist in the Pattern Engine or can be sent by the Pattern Orchestrator. Drools facts are used to satisfy the ’when’-part of the Drools Rules (conditional elements) aiming to execute a rule (action). The execution of a rule, in this case, corresponds to execution of Java code. The Drools Facts that refer to SPDI and QoS properties are those of type Property.

As an example of Drools Rule, Listing 2 shows the specification of QoS (bandwidth) property. The ’when’-part of the rule specifies: the two activity placeholders *pA* and *pB* along with their bandwidth properties (lines 3-6), the link between them along with its corresponding property (lines 7-8), the order (sequence) in which *pA* and *pB* are executed (line 9), and the *PR4* bandwidth property that refers to the sequence. In the ’then’-part, the *PR4* bandwidth property is guaranteed if all the above and the conditions mentioned in the property hold (line 10).

Such a Drools Rule corresponds to a pattern. In this case, a QoS pattern is defined to monitor and enforce a minimum bandwidth. A Pattern Engine equipped with such a pattern, can verify if the *qosbandwidth* property holds for a given IoT application.

### 5.3. Configuring the SDN

Our main objective is to give the IoT application developers easy-to-use tools that enable to define requirements related to the network without having to define detailed network configurations. Hence, our approach allows the automatic generation of network configurations from the initially defined user requirements, which are then translated into patterns via the Pattern Orchestrator (Section 5.1) and converted into facts and rules to be executed by the Pattern Engine (Section 5.2). Finally, the Pattern Engine executes rule actions that implement the network configurations, which we describe in detail in this section.

Listing 2: Specification of QoS (Bandwidth) Property via Drools

rule "Sequence Bandwidth Verification"
when
Placeholder(pA:=placeholderid)
PR1: Property (pA:=subject, category=="qosbandwidth", prvalue1:=value, satisfied==true)
Placeholder(pB:=placeholderid)
PR2: Property (pB:=subject, category=="qosbandwidth", prvalue2:=value, satisfied==true)
Link (rId:=recipeID, orchLink:=linkid)
PR3: Property (rId:=recipeID, orchLink:=subject, category=="qosbandwidth", prvalue3:=value,
     satisfied==true)
SEQ: Sequence(rId:=recipeID, sId:=placeholderid, pA:=placeholdera, pB:=placeholderb,
     orchLink:=orchlink)
PR4: Property (rId:=recipeID, sId:=subject, category=="qosbandwidth", prvalue4:=value,
     prvalue4<=prvalue1, prvalue4<=prvalue2, prvalue4<=prvalue3, satisfied==false)
then
modify(PR4){satisfied=true};
end
			


Listing 3: Call to the SDN Controller for configuring the communication with a switch
*curl − XPUT − d’"tcp :*
     OVS_IP_ADDR:*TCP_L_isten_P_ORT"’http : //*SDN_IP:*SDN_L_isten_P_ORT/v1.0/con f /switches/*Switch_ID/
     ovsdb_addr
			


As shown in the architecture (Figure 1), the Pattern Engine is incorporated with the SDN Controller that has access to one or multiple SDN Switches. After a rule triggers, the Pattern Engine executes a rule action (Java program code), while relying on the functionalities of the SDN Controller. Particularly, we have implemented this configuration through the REST interface of the SDN Controller (we used the RYU controller (https://osrg.github.io/ryu/)). The Pattern Engine can then dynamically adjust the network based on previously received rules. This enables in general a more agile management of the network, as compared to regular SDN controller based approaches.

Before we can add queue settings and QoS rules, the first step to configuring the SDN is conducted during the bootstrapping phase to establish the communication with a switch. Listing 3 shows this first call that informs the SDN controller that the SDN switch (implemented using Open vSwitch (https://www.openvswitch.org/; OVS) is listening on a particular IP address and port number. Then, to communicate between SDN controller and a switch the Open vSwitch Database Management Protocol) (https://tools.ietf.org/html/rfc7047) in conjunction with OpenFlow (https://www.opennetworking.org/images/stories/downloads/sdn-resources/onf-specifications/openflow/openflow-spec-v1.3.1.pdf) version 1.3 are used. Thereby, the *OVS_IP_ADDR* is the IP address of the Open vSwitch, *TCP_Listen_PORT* is the port number of the switch, *SDN_IP* is the address of the SDN controller, *SDN_Listen_PORT* is its port number (for HTTP communication), and *Switch_ID* is given to uniquely identify a switch.

In a second step, a call is made to the SDN Controller to set a queue table for a particular port or all ports of a switch. Thereby, this table comprises the following parameters: *Port_Name* is the name corresponding to the port planned to set queue for, and *linux-htb*/*linux-hfsc* are two options to specify the queuing discipline (representing respective queuing algorithms in the Linux kernel). Further, we need to specify the maximum and minimal rate (*max_rate* and *min_rate* properties), and we can specify the data rate limits for each queue in the queues property. The ID of a particular queue is the index of the queue in the queues list.

Finally, the SDN Controller is called by the Pattern Engine to install QoS flow rules in the flow table at a switch. Each installed QoS flow rule will *match* source and destination IP addresses of a packet with given source and destination IP addresses given. The matched packets will be forwarded to a queue with a particular queue ID, and so the traffic of similar packets will be limited according to the bandwidth limitation specified in that queue.

Listing 4: Call to the SDN Controller for configuring the queues at the switch
*curl − XPOST − d’{”port_n_ame” : ”*Port_Name", "type": "*linux − htb*|linux-hfsc", "max_rate":
     "*int*”, ”*queues*” : [{”*max_r_ate*” : ”int", "min_rate":
     "*int”}, ...]}’http :* //SDN_IP:*SDN_L_isten_P_ORT/qos/queue/*Switch_ID
			


Listing 5: Call to the SDN Controller for configuring a flow entry
*curl − XPOST − d’”priority” : 1, ”match” : ”ipv4_s_rc” : ”10.0.0.1”, ”ipv4_d_st” : ”10.0.0.2”, ”actions” : [”type” : ”ENQUE”, ”queueid” : 1, ”port” : 1]...’http : //*
     SDN_IP:*SDN_L_isten_P_ORT/qos/rules/*Switch_ID
			


The above described approach works not only with multiple SDN switches, but also multiple SDN Controllers could be handled by the Pattern Engine. By simply maintaining the references (switch IDs and SDN Controller IPs) used in the described calls above, this approach allows to manage complex cases of networks with multiple switches and controllers.

## 6. Proof of Concept Application and Evaluation

In the following, we present an application that build up on the presented architecture and implemented components (Section 6.1) and evaluate our approach in context of this application concerning the network performance and semantic reasoning.

### 6.1. Oil Detection Application

**Figure 7:**
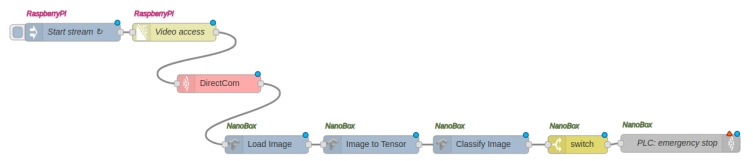
IoT application flow for oil leakage detection.

This application utilizes the developed architecture for automated detection of oil leakages occurring around the inner bearings of wind turbines. This is a problem that can remain unrecognized for too long by the maintenance engineers and an automatic detection is promising for wind park operators. The application flow is implemented in Node-RED and shown in Figure 7. The video stream from the camera is read via the ’video access’ node. It transmits the video stream to an AI pipeline via the *DirectCom* node (Section 4.2 and Figure 3) that enables the definition of application-specific QoS constraints. In this example the video frame rate is specified to a minimum of 15 frames per second (as shown in Figure 3) and configured/monitored by the Pattern Orchestrator and Pattern Engine. The AI pipeline can then load each image frame, transfers it to a tensor and finally classifies the image into two classes (’no oil’ or ’oil’ detected). The image classification is based on a re-trained MobileNet [51] neural network and is implemented using TensorFlow [52]. Finally, the programmable logic controller (PLC) [53] for the wind turbine is triggered in case leaked oil is detected.

**Figure 8:**
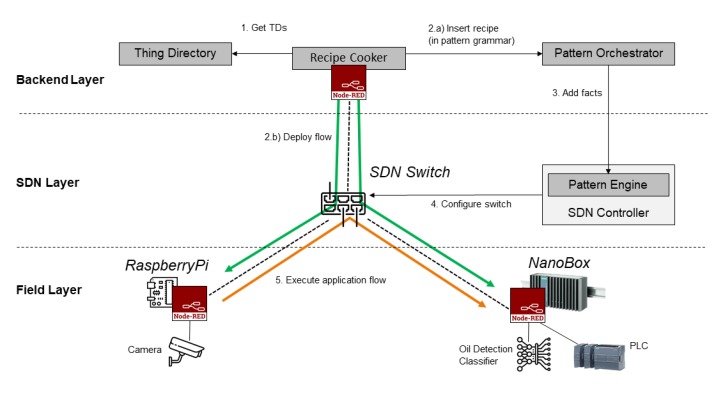
Setup of the oil leakage detection application.

Figure 8 (Icons made by Pause08, Becris, Eucalyp, and freepik from www.flaticon.com; images of NanoBox and PLC are under copyright of Siemens AG) shows the deployment setup of this IoT application flow. The IT infrastructure within the wind turbine is connected via an SDN programmable network. Here, a Raspberry Pi device provides access to the video camera and a Siemens SIMATIC NanoBox [54] is available on the network as an edge resource with extended computing power. First, the Recipe Cooker retrieves the relevant TDs for all registered devices to access their metadata. Then, the distributed application flow is defined in the Recipe Cooker as described above. In the second step, the application flow is translated to patterns (Section 5.1) and transmitted to the Pattern Orchestrator to configure the network accordingly. At the same time, the application flow is deployed using DNR [33], i.e., each node contained in the application is instantiated within the Node-RED environment of the device to which it has been assigned.

### 6.2. Performance Assessment

The subsections below present an initial evaluation of the performance of the key building blocks of the proposed approach.

#### 6.2.1. Evaluation of Network Usage

**Figure 9:**
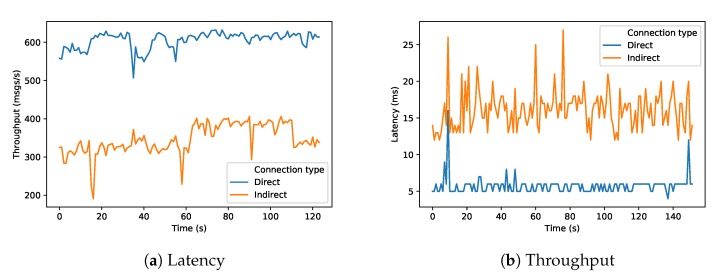
Performance measurements of analysis application in direct vs. indirect mode.

In our experiment (Section 6.1), a live video stream was transmitted between a Raspberry Pi and a NanoBox over a network configured by an SDN controller. In order to evaluate the influence of our approach and particularly the utilization of the DirectCom node and specified QoS in the application flow (see Figure 7), we compared the brokered architecture (as an *indirect* communication using MQTT via the original DNR broker as part of the Recipe Cooker) and the *direct* communication (using UDP with the DirectCom node). To compare the latency, a timestamp packet was sent from Raspberry Pi every one second; once it arrived at the NanoBox, another timestamp was generated and the difference was calculated as latency (or end-to-end delay). We did this procedure for both approaches. The resulting latency measurements over time are presented in Figure 9a. In the graph, it becomes clear that over time the direct communication approach has less latency than the brokered architecture approach. It has been reduced around 50%.

Further, we analyzed the difference in received throughput between the two approaches. To do that, 1000 messages per second were sent from the Raspberry Pi, and every message is about 73 Bytes. In the NanoBox, we checked how many messages were received per second. We did this procedure for both approaches. The measured throughput over time is shown in a graph of Figure 9b. From the graph, we can see that the direct communication approach has better throughput (received messages/second) compared to the brokered architecture approach and it improves by around 50%.

#### 6.2.2. Evaluation of Pattern Engine

As an early verification of the feasibility of the proposed pattern reasoning approach, a proof of concept environment has been setup based on the JBoss Drools Engine v7.15 (https://www.drools.org/download/download.html), and gRPC (https://www.grpc.io/) with Protocol Buffers Version 3 (https://developers.google.com/protocol-buffers/).

In more detail, the testbed setup features a gRPC server is deployed on a desktop system (Core i7, 8GB RAM), loading the Pattern Engine with a basic set of Drools rules. A test client is used to make gRPC calls to the server to request verification of the QoS pattern rule presented in Listing 2 above.

Using the above test setup, and based on the complexity of the modeled IoT environment, i.e., the number of placeholders stored as facts within the Drool knowledge base, the execution time ranges from 19 ms for 10 placeholders to 82 ms for 100 placeholders.

While a more detailed performance evaluation will follow, investigating in more detail the performance impact of modeling more complex environments and supporting and evaluation a larger set of pattern rules, these initial results validate the feasibility of real-time pattern-driven property verification and the timely triggering of needed adaptations.

## 7. Conclusions and Future Work

In this work, we present our approach for networking-aware IoT application development. Our architecture is centered around the Recipe Cooker, a tool based on Distributed Node-RED, that allows the definition of application flows, which we extended to define QoS constraints from application perspective and to provide an integrative view on application and network. We provide then a mechanism that automatically translates these application-specific QoS constraints into network-specific constraints, which are configured and monitored via an SDN controlled network deployment. The description of the application flow is based on a semantic model upon which we conduct the auto-translation into a pattern language for defining facts that are fed into a rule engine.

We applied our approach in an IoT application for oil leakage detection within the bearings of a wind turbine. We demonstrated that the application-specific QoS (e.g., video frame rate of 15 frames per second) are translated into bandwidth constraints that are configured on the SDN controller. We conducted first performance assessments on the network usage resulting from utilization of our components and evaluate the performance of the reasoning in the Pattern Engine.

Our approach is backward compatible with existing Node-RED applications, as DNR is fully backward compatible, and applications can be simply integrated using standard import functionality. When importing an existing IoT application into our system, subsequently, the distribution of application parts onto different devices and their connectivity via DirectCom node can be configured.

Following up on these results, our road map for this research involves multiple directions, e.g., we will leverage on the findings of this work to improve application development for distributed AI based on IoT devices by facilitating the network constraint consideration. Thereby we will not only investigate on improving the inference but also the training of AI (e.g., using federated learning [55]).

## Figures and Tables

**Table 1:** Field names of the Drools Fact object in the addFact request.

Name	Description	Valid Value Example
recipeID	The ID of the recipe the fact belongs to	“WF1”
factID	The identifier of the fact object itself	“WF1-1”
from	Originator of the message	“Orchestrator”
factMessage	The fact itself	“DisplayImage, 80801, PiB”
type	The object type of the fact	“Softwarecomponent”

## References

[1] Raggett D. (2015). The web of things: Challenges and opportunities. Computer.

[2] Hannelius T., Salmenpera M., Kuikka S. Roadmap to adopting OPC UA. Proceedings of the 2008 6th IEEE International Conference on Industrial Informatics.

[3] Swetina J., Lu G., Jacobs P., Ennesser F., Song J. (2014). Toward a standardized common M2M service layer platform: Introduction to oneM2M. IEEE Wirel. Commun..

[4] Pahl M.O., Carle G. (2013). Taking Smart Space Users into the Development Loop: An Architecture for Community Based Software Development for Smart Spaces. Proceedings of the 2013 ACM Conference on Pervasive and Ubiquitous Computing Adjunct Publication.

[5] Charpenay V., Käbisch S., Kosch H. (2016). Introducing Thing Descriptions and Interactions: An Ontology for the Web of Things. SR+ SWIT@ ISWC.

[6] Thuluva A.S., Bröring A., Medagoda G.P., Don H., Anicic D., Seeger J. (2017). Recipes for IoT Applications. Proceedings of the IoT ’17 Seventh International Conference on the Internet of Things.

[7] Seeger J., Deshmukh R.A., Bröring A. Running Distributed and Dynamic IoT Choreographies. Proceedings of the 2018 IEEE Global Internet of Things Summit (GIoTS) Proceedings.

[8] Seeger J., Deshmukh R.A., Sarafov V., Bröring A. (2019). Dynamic IoT Choreographies. IEEE Pervasive Comput..

[9] Seeger J., Bröring A., Pahl M.O., Sakic E. Rule-Based Translation of Application-Level QoS Constraints into SDN Configurations for the IoT. Proceedings of the 2019 European Conference on Networks and Communications (EuCNC).

[10] Sheng Q.Z., Qiao X., Vasilakos A.V., Szabo C., Bourne S., Xu X. (2014). Web services composition: A decade’s overview. Inf. Sci..

[11] Lécué F., Gorronogoitia Y., Gonzalez R., Radzimski M., Villa M. SOA4All: An innovative integrated approach to services composition. Proceedings of the International Conference on Web Services.

[12] Kopecky J., Gomadam K., Vitvar T. hrests: An html microformat for describing restful web services. Proceedings of the WI-IAT’08 International Conference on Web Intelligence and Intelligent Agent Technology.

[13] Mayer S., Verborgh R., Kovatsch M., Mattern F. (2016). Smart Configuration of Smart Environments. IEEE Trans. Autom. Sci. Eng..

[14] El Kaed C., Khan I., Van Den Berg A., Hossayni H., Saint-Marcel C. (2017). SRE: Semantic rules engine for the industrial Internet-of-Things gateways. IEEE Trans. Ind. Inform..

[15] Hayyolalam V., Pourhaji Kazem A.A. (2018). A systematic literature review on QoS-aware service composition and selection in cloud environment. J. Netw. Comput. Appl..

[16] Mokhtar S.B., Preuveneers D., Georgantas N., Issarny V., Berbers Y. (2008). EASY: Efficient semAntic Service discoverY in pervasive computing environments with QoS and context support. J. Syst. Softw..

[17] Moustafa A., Zhang M., Bai Q. (2016). Trustworthy Stigmergic Service Compositionand Adaptation in Decentralized Environments. IEEE Trans. Serv. Comput..

[18] Liu C., Cao J., Wang J. (2017). A Reliable and Efficient Distributed Service Composition Approach in Pervasive Environments. IEEE Trans. Mob. Comput..

[19] Nunes B.A.A., Mendonca M., Nguyen X.N., Obraczka K., Turletti T. (2014). A Survey of Software-Defined Networking: Past, Present, and Future of Programmable Networks. IEEE Commun. Surv. Tutor..

[20] McKeown N., Anderson T., Balakrishnan H., Parulkar G., Peterson L., Rexford J., Shenker S., Turner J. (2008). OpenFlow: Enabling innovation in campus networks. ACM SIGCOMM Comput. Commun. Rev..

[21] Blake S., Black D., Carlson M., Davies E., Wang Z., Weiss W. RFC 2475—An Architecture for Differentiated Services; Technical Report. https://dl.acm.org/doi/pdf/10.17487/RFC2475.

[22] Braden R., Clark D., Shenker S. RFC 1633—Integrated Services in the Internet Architecture: An Overview. https://dl.acm.org/doi/pdf/10.17487/RFC1633.

[23] Mekikis P.V., Ramantas K., Sanabria-Russo L., Serra J., Antonopoulos A., Pubill D., Kartsakli E., Verikoukis C. (2019). NFV-enabled experimental platform for 5G Tactile Internet support in industrial environments. IEEE Trans. Industrial Inform..

[24] Sarrigiannis I., Ramantas K., Kartsakli E., Mekikis P.V., Antonopoulos A., Verikoukis C. (2019). Online VNF Lifecycle Management in a MEC-enabled 5G IoT Architecture. IEEE Internet Things J..

[25] Seeger J., Bröring A., Carle G. (2020). Optimally Self-Healing IoT Choreographies. arXiv.

[26] Naman A.T., Wang Y., Gharakheili H.H., Sivaraman V., Taubman D. (2018). Responsive high throughput congestion control for interactive applications over SDN-enabled networks. Comput. Netw..

[27] Akella A.V., Xiong K. Quality of Service (QoS)-Guaranteed Network Resource Allocation via Software Defined Networking (SDN). Proceedings of the 2014 IEEE 12th International Conference on Dependable, Autonomic and Secure Computing.

[28] Kucminski A., Al-Jawad A., Shah P., Trestian R. QoS-based routing over software defined networks. Proceedings of the 2017 IEEE International Symposium on Broadband Multimedia Systems and Broadcasting (BMSB).

[29] Li F., Cao J., Wang X., Sun Y. (2017). A QoS Guaranteed Technique for Cloud Applications Based on Software Defined Networking. IEEE Access.

[30] Guck J.W., Van Bemten A., Kellerer W. (2017). DetServ: Network Models for Real-Time QoS Provisioning in SDN-Based Industrial Environments. IEEE Trans. Netw. Serv. Manag..

[31] Gorlatch S., Humernbrum T. Enabling high-level QoS metrics for interactive online applications using SDN. Proceedings of the 2015 International Conference on Computing, Networking and Communications (ICNC).

[32] Ur B., Pak Yong Ho M., Brawner S., Lee J., Mennicken S., Picard N., Schulze D., Littman M.L. (2016). Trigger-Action Programming in the Wild: An Analysis of 200,000 IFTTT Recipes. Proceedings of the 2016 CHI Conference on Human Factors in Computing Systems.

[33] Giang N.K., Blackstock M., Lea R., Leung V.C.M. Developing IoT applications in the Fog: A Distributed Dataflow approach. Proceedings of the 2015 5th International Conference on the Internet of Things (IOT).

[34] Salatino M., De Maio M., Aliverti E. (2016). Mastering Jboss Drools 6.

[35] Forgy C.L. (1989). Rete: A fast algorithm for the many pattern/many object pattern match problem. Readings in Artificial Intelligence and Databases.

[36] Bröring A., Schmid S., Schindhelm C.K., Khelil A., Käbisch S., Kramer D., Phuoc D.L., Mitic J., Anicic D., Teniente E. (2017). Enabling IoT Ecosystems through Platform Interoperability. IEEE Softw..

[37] Guha R., Brickley D., Macbeth S. (2016). Schema. org: Evolution of structured data on the web. Commun. ACM.

[38] Hodgson R., Keller P.J. (2011). QUDT-Quantities, Units, Dimensions and Data Types in OWL and XML. http://www.qudt.org.

[39] Shi W., Cao J., Zhang Q., Li Y., Xu L. (2016). Edge computing: Vision and challenges. IEEE Internet Things J..

[40] Meusel R., Bizer C., Paulheim H. A web-scale study of the adoption and evolution of the schema. org vocabulary over time. Proceedings of the 5th International Conference on Web Intelligence, Mining and Semantics.

[41] Fysarakis K., Papoutsakis M., Petroulakis N., Spanoudakis G. Towards IoT Orchestrations with Security, Privacy, Dependability and Interoperability Guarantees. Proceedings of the 2019 IEEE Global Communications Conference (GLOBECOM 2019).

[42] Soultatos O., Papoutsakis M., Fysarakis K., Hatzivasilis G., Michalodimitrakis M., Spanoudakis G., Ioannidis S. Pattern-Driven Security, Privacy, Dependability and Interoperability Management of IoT Environments. Proceedings of the 2019 IEEE 24th International Workshop on Computer Aided Modeling and Design of Communication Links and Networks (CAMAD).

[43] Fysarakis K., Spanoudakis G., Petroulakis N., Soultatos O., Bröring A., Marktscheffel T. Architectural Patterns for Secure IoT Orchestrations. Proceedings of the Global IoT Summit 2019 (GIoTS’19).

[44] Pino L., Spanoudakis G., Fuchs A., Gurgens S. Discovering Secure Service Compositions. Proceedings of the 4th International Conference on Cloud Computing and Services Sciences (CLOSER 2014).

[45] Pino L., Spanoudakis G., Krotsiani M., Mahbub K. (2017). Pattern Based Design and Verification of Secure Service Compositions. IEEE Trans. Serv. Comput..

[46] Maña A., Damiani E., Guergens S., Spanoudakis G. Extensions to Pattern Formats for Cyber Physical Systems. Proceedings of the 31st Conference on Pattern Languages of Programs (PLoP’14).

[47] Petroulakis N.E., Spanoudakis G., Askoxylakis I.G. Fault tolerance using an sdn pattern framework. Proceedings of the IEEE Global Communications Conference (GLOBECOM).

[48] Extended Backus-Naur Form. https://tomassetti.me/ebnf.

[49] ANother Tool for Language Recognition. https://www.antlr.org.

[50] Business Rules Management System (BRMS). https://www.drools.org.

[51] Howard A.G., Zhu M., Chen B., Kalenichenko D., Wang W., Weyand T., Andreetto M., Adam H. (2017). Mobilenets: Efficient convolutional neural networks for mobile vision applications. arXiv.

[52] Abadi M., Agarwal A., Barham P., Brevdo E., Chen Z., Citro C., Corrado G.S., Davis A., Dean J., Devin M. (2016). Tensorflow: Large-scale machine learning on heterogeneous distributed systems. arXiv.

[53] Berger H. (2012). Automating with SIMATIC: Controllers, Software, Programming, Data.

[54] Siemens SIMATIC Box IPC. https://new.siemens.com/global/en/products/automation/pc-based/simatic-box-ipc.html#SIMATICIPC227E.

[55] Park J., Samarakoon S., Bennis M., Debbah M. (2019). Wireless network intelligence at the edge. Proc. IEEE.

